# Lumbar disc space height in relation to neural foraminal dimensions and patient characteristics: A morphometric analysis from L1-S1 using computed tomography

**DOI:** 10.1016/j.bas.2024.104162

**Published:** 2024-12-10

**Authors:** David Shin, Ethan Vyhmeister, Daniel Im, Andrew Fay, Owen Faehner, Andrew Cabrera, Alexander Bouterse, Lauren Seo, Derran Bedward, Mei Carter, Davis Carter, Jacob Razzouk, Omar Ramos, Nathaniel Wycliffe, Wayne Cheng, Olumide Danisa

**Affiliations:** a School of Medicine, Loma Linda University, Loma Linda, CA, USA; b Department of Orthopaedic Surgery, Duke University Health System, Durham, NC, USA; c Twin Cities Spine Center, Minneapolis, MN, USA; d Department of Radiology, Loma Linda University Health, Loma Linda, CA, USA; e Division of Orthopaedic Surgery, Jerry L. Pettis VA Medical Center, Loma Linda, CA, USA; f Departments of Orthopaedic Surgery and Neurological Surgery, Duke University Health System, Durham, NC, USA

**Keywords:** Computed tomography, Disc space height, Ethnicity, Neuroforamina, Anatomic, Intervertebral disc space

## Abstract

**Introduction:**

The normative relationship between lumbar intervertebral disc space height (DSH) and neuroforaminal dimensions (NFD) has yet to be defined.

**Research question:**

The purpose of this study was to investigate the relationship between lumbar DSH and NFD using computed tomography (CT), accounting for influences of patient demographic and anthropometric characteristics.

**Materials and methods:**

We analyzed CT imaging of 350 female and 350 male patients. Anterior, middle, and posterior DSH were measured. NFD were defined as sagittal anterior-to-posterior (AP) width, axial AP width, foraminal height, and area. Statistical analyses were performed to assess associations among DSH, NFD, and patient height, weight, body mass index, sex, and ethnicity.

**Results:**

Irrespective of disc level, mean anterior, middle, and posterior DSH were 7.98 mm (n = 3500), 8.16 mm (n = 3500), and 4.09 mm (n = 3500). DSH measurements demonstrated increasing, linear trends moving caudally from L1-L2 to L5-S1, while NFD demonstrated a unimodal distribution pattern with largest NFD at L3-L4 and smallest NFD at L1-2 and L5-S1. Male patients demonstrated larger DSH compared to female patients from L1-S1. Asian patients demonstrated taller DSH across all levels L1-S1.

**Discussion and conclusion:**

This study describes 38,500 CT-based L1-S1 DSH and NFD in young patients without spinal pathology. DSH follows an increasing trend moving caudally from L1-S1, while NFD demonstrate a unimodal distribution clustered at L3-L4. NFD are not moderately or strongly associated with DSH. DSH is influenced by sex and ethnicity but is not moderately or strongly influenced by patient height, weight, and BMI.

## Highlights

• Disc space height (DSH) follows an increasing trend moving caudally from L1-S1.
• Neuroforaminal dimensions (NFD) showed a unimodal distribution clustered at L3-L4.
• NFD are not moderately or strongly associated with DSH.
• DSH is influenced by sex and ethnicity but not by patient height, weight, and BMI.

## 1 Introduction

Thorough understanding of lumbar morphometry is essential during procedural intervention for lumbar decompression, stabilization, and correction of deformity. In particular, comprehensive knowledge of lumbar intervertebral disc space height (DSH) cannot be overstated given its importance for diagnostic interpretations. This also holds true when surgical techniques are used to reconstruct or recreate the disc space height as in the case of appropriate cage selection during interbody fusion (Hollinshead, 1965; Keller et al., 2005; Pellet et al., 2011). Lumbar DSH has been well-studied using the imaging of healthy cohorts in an effort to provide normative parameters for use in the preoperative assessment of fusion candidates (Roberts et al., 1997; Mansur et al., 2020). Radiographic measurements of DSH in relation to other anatomic considerations such as lumbar sagittal alignment have also been described (Zhang et al., 2018).

The relationship between DSH and neuroforaminal height (NFH) has been well studied and seems intuitive, specifically, that DSH correlates with NFH (Lin et al., 2021; Hasegawa et al., 1995; Na et al., 2012; Wang et al., 2007; Cinotti et al., 2002; Karabekir et al., 2011). The normative relationship between DSH and neuroforaminal dimensions (NFD), however, has yet to be defined. The neuroforaminal dimensions consist of the sagittal anterior-to-posterior (AP) width, axial AP width, NFH, and foraminal area (Cramer et al., 2003; Senoo et al., 2014; Lee et al., 2010). Understanding the relationship between DSH and NFD may aid in establishing quantitative, and normative baselines for several applications including but not limited to: device design and implementation, comprehension of the concepts of direct and indirect surgical decompression, correction of sagittal and coronal balance, restoration of disc height, and appropriate cage selection to avoid distractive and compressive forces on the vertebral endplates. Taking into consideration patient demographic and anthropometric characteristics, however, is important during analysis of DSH and NFD morphometry to account for the diversity of patient presentations (Acharya et al., 2010).

This study is the second phase of the Harianja et al. study (Harianja et al., 2023). In the Harianja et al. study, we investigated the normative NFD in 600 young patients without spinal pathology. The objective of this second phase is to characterize the relationship between NFD and DSH from L1-S1 using computed tomography (CT) of young patients free of spinal pathology, accounting for influences of patient demographic and anthropometric characteristics.

## 2 Methods

### 2.1 Data collection

Following IRB approval (#5220055), we evaluated the medical and radiographic records of patients between 18 and 35 years of age who received abdomen and pelvis, or lumbar CT (GE Discovery 750 HD 64 slice CT Scanner) between February of 2018 and March of 2023. Patient consent was not required due to the nature of this retrospective, radiographic study. The charts of all patients considered for inclusion within this study were reviewed in a systematic, consecutive manner corresponding to the chronological sequence in which their imaging was completed. To establish an equal number of male and female patients, the charts of male and female candidates were separated and reviewed in an alternating manner. Those with a history of degenerative disc disease, scoliosis (defined as a coronal deformity greater than 10°), congenital lumbar stenosis (CLS), spondylolisthesis, spinal trauma, neoplasm, existing spinal hardware, had CT imaging performed due to back pain, or previous spinal surgery were excluded from this study. A patient was identified as having CLS if any of their L2-L5 pedicle lengths were smaller than the thresholds described by Singh et al. in their prospective study of CLS (Singh et al., 2005).

**Fig. 1:**
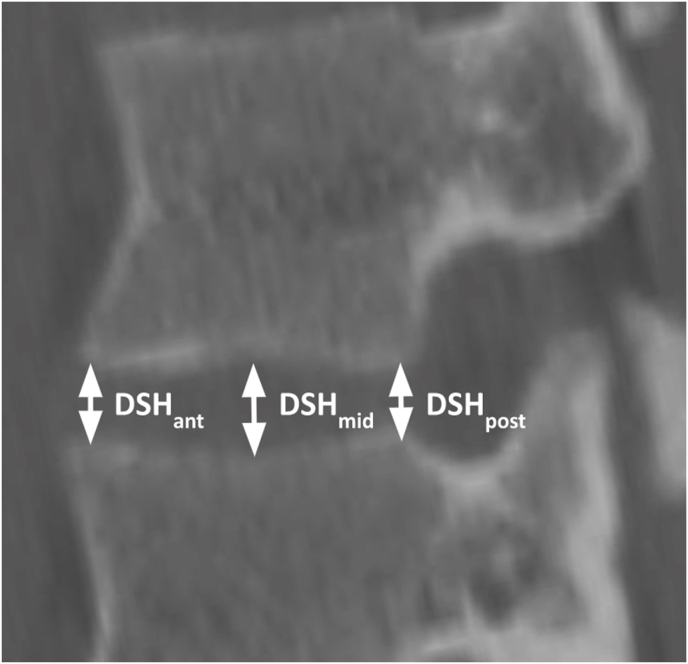
Dsh Measurements Measurement technique of anterior, middle, and posterior disc space height.

**Fig. 2:**
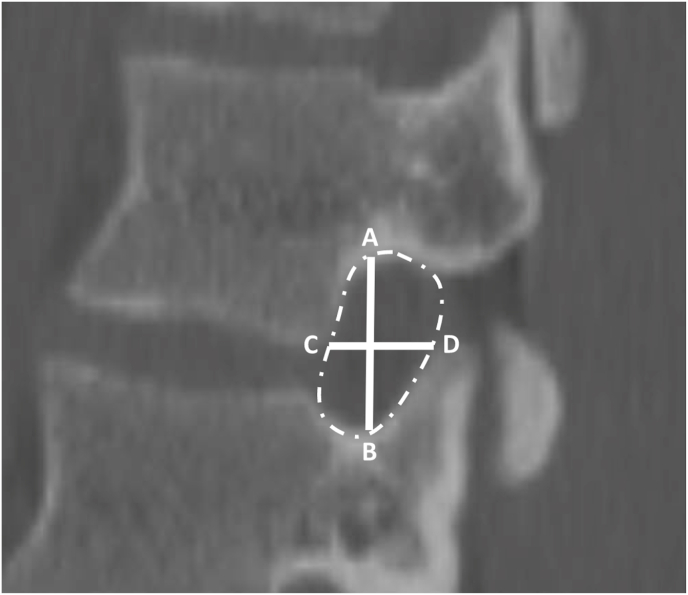
NFD Measurements NFH was measured as illustrated by line, NFH was measured as illustrated by line AB. Sagittal AP width was measured as illustrated by line CD. Axial AP width was measured with the same methodology, though in the axial view. Foraminal area was measured as illustrated by the foramen contained within the hatched line.

All images were reviewed and measured using the IMPAX6 (Agfa-Gavaert, Mortsel, Belgium) picture archiving and communication system with a window designation of 2000 Hounsfield Unit and a level designation of 500 Hounsfield Unit. All measurements were performed by four medical students (JR, EV, MC, DC) trained by a board-certified neuroradiologist (NW) to measure anterior, middle, and posterior DSH and bilateral L1-S1 NFD, defined as the following: sagittal anterior-to-posterior (AP) width, axial AP width, NFH, and neuroforaminal area. Fig. 1 provides an illustration of the measurement technique for DSH. Fig. 2 provides an illustration of the measurement technique for NFD.

Anterior and posterior disc space heights were obtained from the sagittal view in millimeters (mm) and were measured as the shortest distances between the planes of the anterior and posterior endplates as described by Harianja et al. (2023) Middle DSH was measured at the center of each interspace as similarly described by Harianja et al. (see Fig. 1). The maximal distances between the superior vertebral body posterior margin and the inferior articular facet anterior margin defined axial and sagittal AP dimensions. NFH was defined as the maximum distance between the inferior notch of the pedicle of the superior vertebral body and the superior notch of the pedicle of the inferior vertebral body (see Fig. 2). Area of the neuroforamen was measured from the sagittal view using IMPAX6 tracing freeform tool. Patient demographic and anthropometric characteristics—sex, race, ethnicity, age, body mass index (BMI), height, and weight—were also collected, with all data collection performed using Microsoft Excel version 16.58 (Microsoft Corporation, 2022; Redmond, WA, USA).

### 2.2 Statistical and power analyses

All statistical analyses were performed using SPSS version 28 (IBM Corporation, 2021; Armonk, NY, USA) with an alpha setting of .05 used to denote statistical significance. Levene's homogeneity of variance test and regression residual plots were employed to assess homoscedasticity (Parra-Frutos, 2013). Kolmogorov-Smirnov tests and Q-Q plots were used to assess normality of data distribution (Vetter, 2017a, 2017b). Correlation coefficients were classified as weak, moderate, and strong corresponding to value ranges of 0–.4, .4–.7, and .7–1, respectively (Ratner, 2009; Razzouk et al., 2022). To assess associations among radiographic (DSH and NFD), demographic, and anthropometric variables, enter-method multivariate linear regression models with zero-order and partial correlations were analyzed. Differences in DSH based on sex, race, and ethnicity were assessed using two-way analysis of covariance (ANCOVA) with anthropometric covariates, estimated marginal means, and type III sum-of-square specification. Measurement differences in NFD and DSH among disc levels were analyzed using one-way analysis of variance (ANOVA) with *post-hoc* Bonferroni and Tukey corrections.

To assess for sufficient sample size, nondirectional partial correlation power analysis was conducted with a sample size of 700, specified parameters of .05 for alpha, a null value of zero, a partial correlation parameter of .300, and an assumed number of four variables to be partialled out (Razzouk et al., 2022). Power analysis determined an achieved power of 100%. Similarly, a univariate linear regression power analysis estimating the power of type III *F*-test was conducted with a sample size of 700, an alpha of .05, a population multiple partial correlation of .300, six total predictors, and four test predictors, with an achieved power of 100%. Finally, one-way ANOVA power analysis was conducted with specified parameters of .05 for alpha, an assumed standard deviation of 1.0, and sample groups of 297, 261, 70, and 60 (corresponding to the ethnic subgroups sample sizes of our study), and group means of 5.48, 5.80, 5.67, and 6.48, with a similar achieved power of 100%.

## 3 Results

### 3.1 Patient characteristics

This study screened a total of 809 patients, of which 109 were excluded. Of those excluded, 41 possessed poor or insufficient imaging, 37 had previous spinal surgery, 11 demonstrated scoliosis, 7 had congenital lumbar stenosis, 6 had spondylolisthesis, and 7 had lumbar vertebral fracture. Of the 700 patients vetted for inclusion within the study, 350 were male and 350 were female. Male versus female values with respect to age, BMI, height, and weight are reported in Table 1. With respect to ethnicity, 42.43% (n = 297) of patients were Hispanic, 37.29% (n = 261) were Caucasian, 10.00% (n = 70) were African-American, 8.57% (n = 60) were Asian, and 1.57% (n = 11) were listed as other. From the 700 patients, 38,500 measurements were performed: 40 NFD and 15 DSH measurements across L1-S1 per patient.

**Table 1:** Patient characteristics (n = 700).

Characteristic	Male Mean (SD) n = 350	Female Mean (SD) n = 350	*p*
Age	27.31 (5.21)	28.53 (5.06)	**.005**
BMI (kg/m^2^)	28.37 (7.20)	28.94 (7.84)	.336
Height (m)	1.76 (.08)	1.63 (.09)	**<.001**
Weight (kg)	88.52 (24.79)	76.69 (21.76)	**<.001**

### 3.2 Disc space height

Without differentiating between L1-S1 disc levels, mean anterior, middle, and posterior disc space heights were 7.98 mm (n = 3500), 8.16 mm (n = 3500), and 4.09 mm (n = 3500). Table 2 displays mean anterior, middle, and posterior DSH values per disc level. All DSH measurements demonstrated a statistically significant trend of increasing in size moving caudally from L1-L2 to L4-L5 (see Table 3). Moving from L4-L5 to L5-S1, anterior DSH continued to increase, while the middle and posterior DSH decreased in height.

**Table 2:** Mean (SD) disc space height (mm) from L1-S1 (n = 700).

Disc Level	Anterior	Middle	Posterior
L1-L2	5.68 (1.86)	6.61 (1.39)	3.11 (1.15)
L2-L3	6.53 (1.90)	7.90 (1.59)	4.02 (1.24)
L3-L4	7.72 (1.94)	8.83 (1.56)	4.66 (1.38)
L4-L5	9.27 (2.17)	9.31 (1.75)	4.88 (1.45)
L5-S1	10.76 (2.72)	8.14 (1.90)	3.80 (1.41)

**Table 3:** Comparison of disc space height across disc levels.

Disc Space Height	Level of Reference	Level of Comparison	Mean Difference (Reference – Comparison)	*p*	95% Confidence Interval	
					Lower Bound	Upper Bound
*Anterior*	L1-L2	L2-L3	−.83	**<.001**	−1.14	−.52
		L3-L4	−2.02	**<.001**	−2.33	−1.72
		L4-L5	−3.5	**<.001**	−3.81	−3.19
		L5-S1	−5.06	**.003**	−5.36	−4.75
	L2-L3	L3-L4	−1.2	**<.001**	−1.51	−.89
		L4-L5	−2.67	**<.001**	−2.98	−2.36
		L5-S1	−4.23	**<.001**	−4.54	−3.92
	L3-L4	L4-L5	−1.47	**<.001**	−1.78	−1.17
		L5-S1	−3.03	**<.001**	−3.34	−2.72
	L4-L5	L5-S1	−1.56	**<.001**	−1.87	−1.25
*Middle*	L1-L2	L2-L3	−1.29	**<.001**	−1.54	−1.04
		L3-L4	−2.22	**<.001**	−2.47	−1.97
		L4-L5	−2.7	**<.001**	−2.95	−2.46
		L5-S1	−1.53	**<.001**	−1.78	−1.28
	L2-L3	L3-L4	−.93	**<.001**	−1.18	−.68
		L4-L5	−1.41	**<.001**	−1.66	−1.17
		L5-S1	−.24	.062	−.49	.01
	L3-L4	L4-L5	−.49	**<.001**	−.73	−.24
		L5-S1	.69	**<.001**	.44	.93
	L4-L5	L5-S1	1.17	**<.001**	.92	1.42
*Posterior*	L1-L2	L2-L3	−.91	**<.001**	−1.1	−.71
		L3-L4	−1.52	**<.001**	−1.72	−1.33
		L4-L5	−1.69	**<.001**	−1.88	−1.49
		L5-S1	−.66	**<.001**	−.86	−.47
	L2-L3	L3-L4	−.61	**<.001**	−.81	−.42
		L4-L5	−.78	**<.001**	−.97	−.58
		L5-S1	.24	**.004**	.05	.44
	L3-L4	L4-L5	−.17	.174	−.36	.03
		L5-S1	.86	**<.001**	.66	1.05
	L4-L5	L5-S1	1.02	**<.001**	.83	1.22

With respect to DSH measurement differences based on sex—when accounting for race and ethnicity, weight, height, and BMI—DSH values still significantly differed between males and females, with males demonstrating larger DSH measurements for all levels L1-S1 (see Table 4). With respect to DSH measurement differences based on race and ethnicity—when accounting for weight, height, BMI, and sex—no difference in middle DSH values were observed among African American, Hispanic, Caucasian, and Asian patients. Anterior and posterior DSH measurements, however, demonstrated that Asian patients possessed significantly larger DSH values compared to African American, Hispanic, and Caucasian subgroups (*p* = .014). Among African American, Hispanic, and Caucasian subgroups, no differences in DSH values were observed (see Table 5).

**Table 4:** Mean sex differences in disc space height (mm) accounting for weight, height, BMI, and ethnicity.

Disc Level	Anterior			Middle			Posterior		
	Male	Female	*p*	Male	Female	*p*	Male	Female	*p*
L1-2	6.48 (1.81)	4.89 (1.53)	**<.001**	6.86 (1.49)	6.36 (1.24)	**<.001**	3.25 (1.19)	2.98 (1.10)	**<.001**
L2-3	7.23 (1.83)	5.83 (1.71)	**<.001**	8.19 (1.66)	7.61 (1.48)	**<.001**	4.21 (1.23)	3.82 (1.21)	**<.001**
L3-4	8.32 (1.94)	7.12 (1.76)	**<.001**	9.13 (1.53)	8.53 (1.48)	**<.001**	4.88 (1.35)	4.44 (1.38)	**<.001**
L4-5	9.82 (2.16)	8.69 (2.02)	**<.001**	9.62 (1.65)	8.99 (1.80)	**<.001**	5.04 (1.39)	4.73 (1.49)	**.004**
L5-S1	11.14 (2.76)	10.38 (2.64)	**<.001**	8.21 (1.94)	8.07 (1.86)	.311	3.83 (1.45)	3.77 (1.36)	.606

**Table 5:** Mean ethnic and racial differences in disc space height (mm) accounting for weight, height, BMI, and sex.

Disc Level	Anterior Disc Space Height				
	White	Hispanic	Black	Asian	*p*
L1-2	5.80 (1.91)	5.48 (1.76)	5.67 (1.64)	6.48 (1.92)	**.003**
L2-3	6.48 (1.78)	6.49 (1.84)	6.21 (1.73)	7.31 (1.80)	**.008**
L3-4	7.78 (1.91)	7.62 (1.84)	7.50 (1.99)	8.41 (1.94)	**.032**
L4-5	9.31 (2.29)	9.21 (2.08)	9.22 (2.10)	9.44 (2.24)	**.022**
L5-S1	10.68 (2.85)	10.70 (2.35)	10.46 (2.85)	11.64 (2.49)	**.036**
**Middle Disc Space Height**					
L1-2	6.63 (1.44)	6.54 (1.38)	6.49 (1.21)	6.92 (1.43)	.310
L2-3	7.81 (1.48)	7.92 (1.53)	7.83 (1.87)	7.99 (1.85)	.278
L3-4	8.86 (1.54)	8.78 (1.55)	8.62 (1.76)	8.81 (1.76)	.365
L4-5	9.34 (1.77)	9.33 (1.66)	9.14 (1.71)	9.25 (2.18)	.836
L5-S1	8.00 (1.21)	8.21 (1.72)	7.96 (2.33)	8.55 (2.21)	.158
**Posterior Disc Space Height**					
L1-2	3.09 (1.24)	3.03 (1.05)	3.25 (1.18)	3.64 (1.07)	**.007**
L2-3	3.80 (1.25)	4.10 (1.14)	4.08 (1.20)	4.69 (1.45)	**<.001**
L3-4	4.40 (1.39)	4.79 (1.23)	4.59 (1.48)	5.29 (1.67)	**<.001**
L4-5	4.70 (1.42)	4.93 (1.25)	4.63 (1.26)	5.09 (.97)	**.036**
L5-S1	3.61 (1.43)	3.94 (1.32)	3.65 (1.63)	4.24 (1.30)	**.002**

### 3.3 Disc space height in relation to neural foraminal dimensions

Unimodal distributions were observed with respect to neural foraminal area and NFH, with the smallest areas and NFH measurements observed at L1-L2 and L5-S1, and the largest values observed at L3-L4. Sagittal and axial AP widths, however, followed trends opposite to those of the DSH measurements: NFD width steadily decreased moving from L1-L2 to L5-S1. All left- and right-sided NFD measurements per disc level are reported in Table 6.

**Table 6:** Mean (SD) L1-S1 neural foraminal dimensions (mm).

Disc Level	Left				Right			
	Sagittal AP	Axial AP	NFH	Area	Sagittal AP	Axial AP	NFH	Area
L1-L2	9.52 (2.12)	9.34 (2.21)	17.31 (2.41)	132.47 (35.18)	9.69 (2.06)	9.30 (2.21)	17.26 (2.27)	132.04 (34.20)
L2-L3	9.24 (2.03)	9.28 (2.32)	18.38 (2.26)	140.54 (37.26)	9.43 (1.99)	9.31 (2.22)	18.77 (3.68)	142.53 (38.06)
L3-L4	9.18 (2.19)	8.99 (2.29)	18.42 (2.48)	143.83 (40.16)	9.20 (2.14)	9.07 (2.25)	18.49 (3.90)	143.89 (41.09)
L4-L5	8.68 (2.13)	8.58 (2.22)	17.81 (2.61)	138.03 (38.92)	8.68 (2.23)	8.60 (2.27)	17.64 (2.40)	137.10 (36.92)
L5-S1	8.81 (2.52)	8.72 (2.60)	15.33 (2.26)	124.38 (36.95)	9.03 (2.44)	8.97 (2.53)	15.14 (2.41)	124.93 (34.03)

Assessing the associations between DSH and NFD values when accounting for patient height, weight, BMI, sex, and ethnicity, zero moderate or strong correlations were observed between any DSH and NFD measurement. NFH, however, demonstrated a statistically significant weak correlation with anterior, middle, and posterior DSH measurements. Assessing the associations between DSH and patient anthropometric characteristics, zero moderate or strong correlations were observed between any DSH and anthropometric factor. Patient weight, however, demonstrated a consistent, weak correlation with DSH measurements for all levels L1-S1. Table 7 displays the enter-method correlation matrix between all DSH and NFD measurements, and all correlations between DSH and anthropometric factors, accounting for patient demographic characteristics.

**Table 7:** Correlation matrix between disc space height and neural foraminal dimensions accounting for height, weight, BMI, sex, and ethnicity^a^.

	Left Sagittal	Left Axial	Left NFH	Left Area	Right Sagittal	Right Axial	Right NFH	Right Area	Height	Weight	BMI
***L1-2 Anterior***	.010	.030	**.119**	.030	.040	.040	**.159**	.030	.000	**.354**	**.193**
***L1-2 Middle***	.060	.070	**.089**	**.099**	.060	.070	**.128**	**.116**	.050	**.214**	**.148**
***L1-2 Posterior***	**.121**	**.127**	**.121**	**.075**	**.088**	**.159**	**.157**	**.113**	.010	**.128**	.060
***L2-3 Anterior***	**.095**	.020	**.199**	**.146**	**.107**	**.108**	.050	**.161**	.040	**.326**	**.194**
***L2-3 Middle***	.070	.030	**.177**	**.149**	.050	.060	.010	**.169**	.040	**.218**	**.134**
***L2-3 Posterior***	**.087**	.040	**.242**	**.161**	.060	.060	**.098**	**.129**	.020	**.274**	**.190**
***L3-4 Anterior***	**.085**	.080	**.214**	**.159**	**.081**	.070	**.124**	**.161**	.020	**.319**	**.206**
***L3-4 Middle***	.000	.020	**.216**	**.126**	.000	.010	**.082**	**.109**	.010	**.219**	**.138**
***L3-4 Posterior***	.060	.070	**.280**	**.144**	.030	.060	**.205**	**.127**	.000	**.307**	**.251**
***L4-5 Anterior***	**.191**	**.199**	**.287**	**.251**	**.184**	**.194**	**.224**	**.208**	.030	**.217**	**.135**
***L4-5 Middle***	.040	.050	**.265**	**.147**	**.090**	**.103**	**.215**	**.174**	.030	**.130**	.060
***L4-5 Posterior***	.050	.040	**.301**	**.111**	.070	.060	**.308**	**.107**	.020	**.271**	**.235**
***L5-S1 Anterior***	.050	.060	.060	.030	.070	**.090**	.070	.060	.020	**.130**	**.105**
***L5-S1 Middle***	.010	.010	**.091**	.000	.030	.050	**.087**	.020	.010	.050	.040
***L5-S1 Posterior***	.020	.040	**.118**	.050	.020	.040	**.236**	.050	.030	**.174**	**.163**

a Values denoted in bold indicate an associated *p*-value <.05.

## 4 Discussion

To the authors' knowledge, this is the first study to date measuring intervertebral disc space height in relation to neural foraminal dimensions while accounting for patient demographic and anthropometric characteristics. Surprisingly, this study found no strong—or even moderate— associations between any DSH and NFD measurement from L1-S1. While DSH was weakly associated with the height of the neuroforamina, it appears that DSH is only a minor influencing factor of foraminal composition. Neuroforamen are a complex three-dimensional space, influenced by a variety of possible structural and biomechanical factors. Such factors include pedicle dimensions, facet joint orientation, ligamentous structures, and dynamic changes from movement and aging. While one may intuit that DSH would directly affect NFD, given the influence of DSH on the angulation and separation of the superior and inferior vertebral bodies, our study's lack of correlation may reflect that NFD are likely influenced by multiple interacting anatomical structures rather than DSH alone. A possible explanation for our findings may be the greater, more direct influence of vertebral morphometry on the neuroforamina such as pedicular length and width, which directly define the size and shape of neuroforamina.

Regarding intervertebral disc height, the DSH measurements obtained in this study are in congruence with the literature, with the L4-L5 DSH being the tallest (Hong et al., 2010; Onishi et al., 2019). This observation may be attributed to the greater spinal mobility at the L4-L5 region (Tibrewal and Pearcy, 1985; Demir et al., 2018). A decrease in DSH at L5-S1 has also been a similar finding across various study populations, likely explained by the L5-S1 disc shape, interface with the sacrum, and greater sustained axial loads (Humzah and Soames, 1988; Urquhart et al., 2014; Schmitt et al., 2004; Oztürk et al., 2008). The observation that middle DSH did not demonstrably increase from L1-S1 in contrast with anterior and posterior DSH may be explained by the relative elliptization of the middle DSH compared to the more rectangular cross sections of the anterior and posterior DSH (Roberts et al., 1997; Luoma et al., 2001; Pfirrmann et al., 2006; Shao et al., 2002).

In our analysis of patient demographic characteristics, male anterior DSH measurements at each level were approximately 1 mm larger compared to female measurements. These observations of greater male versus female DSH are consistent with the findings of Bach et al. in their analysis of 240 CT scans, though when comparing their measurements from participants in the second and third decade of life, our values do vary (Bach et al., 2018). This may be due to the differences in study design and analysis as our study accounted for confounding due to patient demographic and anthropometric factors as well as included a larger sample size. Regarding race and ethnicity, previous radiographic studies on disc space measurements have been performed among several populations including Korean, Pakistani, Nepalese, and Iranian subgroups (Mansur et al., 2020; Hong et al., 2010; Mirab et al., 2018; Fetouh, 2015; Alam et al., 2014). This radiographic study, however, is the first to evaluate DSH and NFD dimensions in Hispanic and African-American cohorts. While no differences in DSH were observed among Caucasian, Hispanic, and African-American subgroups, when these groups were compared to an Asian subgroup, anterior and posterior DSH measurements were significantly taller in the Asian subgroup across all levels L1-S1. These observations highlight the importance of considering differences in DSH based on the demographic background of the patient.

Considering anthropometric characteristics, however, our findings suggest that in the normal, asymptomatic population, height and weight are neither positively nor negatively associated with DSH. Surprisingly, many of the previous studies on radiographic DSH measurements do not account for patient anthropometric or demographic characteristics (Mansur et al., 2020; Hong et al., 2010; Onishi et al., 2019; Alam et al., 2014; Zhou et al., 2000). Nevertheless, the CT-based findings of Bach et al. are congruent with our findings regarding the null association between height or weight, and DSH. As Bach et al. describe the lack of considerable literature on normative DSH values is surprising given DSH restoration is often a key objective during fusion. This problem is only exacerbated by the current paucity of literature regarding implant size selection.

## 5 Limitations

The findings of our study must be viewed in proper context. Given its nature as a single-institution based study, results may not be as generalizable to certain populations due to potential confounding variables unable to be accounted for in this study. Moreover, it is important to qualify that this study is solely composed of patients between 18 and 35 years of age without spinal pathology. Our reasoning for this methodology was to promote the creation of a representative model of “normal” anatomy, though we recognize that defining normal anatomy is subjective. Nevertheless, we tried to be as logical as possible in our definition of “normal” so as to provide reference values that may be equidistant in their application to a specific patient presentation. In this way, a surgeon may use the values in this study as frames of reference that may be adjusted based on individual patient presentations. Additionally, while relevant spinal pathology was identified based on previous diagnosis in either the patients radiographic or electronic medical records, patients often have asymptomatic issues that are not formally diagnosed or documented. As such, the absence of lumbar pathology in all participants was not confirmed through standardized questionnaires or individualized medical history assessments, and undocumented or undiagnosed spinal pathology in our patient population may have affected our findings.

A further limitation of this study is the possibility of measurement error due to subjectivity in reviewer measurements, though we strove to reduce this potential error as much as possible with well-defined measurement protocol and training, determining an acceptable reliability of measurements with an ICC of .842. While some may contend that CT-based morphometry is unable to detect disc herniation or ligamentous infiltration of the neuroforamina, given our patient sample there is limited concern for this potential confounding. CT is advantageous because it is devoid of the ligamentous signals and perineural fat present on MRI and offers sharp display of the bony anatomy.

## 6 Conclusion

This study describes 38,500 CT-based L1-S1 disc space height and neuroforaminal measurements in young patients free of spinal pathology. Normative values of disc space height follow an increasing trend moving caudally from L1-L5, while foraminal dimensions demonstrate a unimodal distribution clustered at L3-L4. Neuroforaminal dimensions are not moderately or strongly associated with disc space height. Disc space height is influenced by sex and ethnicity but is not moderately or strongly influenced by patient anthropometric factors. These conclusions however are generalizable only in a young and asymptomatic cohort. To see if the same holds true for other groups, we plan further study on older, symptomatic (for back and or leg pain) patients, and those with demonstrable spinal pathology.

## Financial support

David Shin, Ethan Vyhmeister, Daniel Im, Andrew Fey, Andrew Cabrera, Alex Bouterse, Owen Faehner, Lauren Seo, Derran Bedward, Mei Carter, Davis Carter, Jacob Razzouk, Omar Ramos, Nathaniel Wycliffe, Wayne Cheng, and Olumide Danisa have nothing to disclose.

## Declaration of competing interest

The authors declare that they have no known competing financial interests or personal relationships that could have appeared to influence the work reported in this paper.
